# Acetylation of Drosha on the N-Terminus Inhibits Its Degradation by Ubiquitination

**DOI:** 10.1371/journal.pone.0072503

**Published:** 2013-08-29

**Authors:** Xiaoli Tang, Sicheng Wen, Dong Zheng, Lynne Tucker, Lulu Cao, Dennis Pantazatos, Steven F. Moss, Bharat Ramratnam

**Affiliations:** 1 Laboratory of Retrovirology, Division of Infectious Diseases, Department of Medicine, Warren Alpert Medical School of Brown University, Providence, Rhode Island, United States of America; 2 COBRE Center for Cancer Research, Division of Hematology and Oncology, Rhode Island Hospital, Warren Alpert Medical School of Brown University, Providence, Rhode Island, United States of America; 3 Division of Gastroenterology, Department of Medicine, Warren Alpert Medical School of Brown University, Providence, Rhode Island, United States of America; Universidad de Granada, Spain

## Abstract

The RNase III enzyme Drosha initiates microRNA (miRNA) biogenesis in the nucleus by cleaving primary miRNA transcripts into shorter precursor molecules that are subsequently exported into the cytoplasm for further processing. While numerous disease states appear to be associated with aberrant expression of Drosha, the molecular mechanisms that regulate its protein levels are largely unknown. Here, we report that ubiquitination and acetylation regulate Drosha protein levels oppositely. Deacetylase inhibitors trichostatin A (TSA) and nicotinamide (NIA) increase Drosha protein level as measured by western blot but have no effects on its mRNA level in HEK293T cells. TSA increases miRNA-143 production in a miRNA sensor assay and in a qPCR analysis in HEK293T cells. Treatment of AGS and HEK293T cells with proteasome inhibitors MG132 or Omuralide increases Drosha protein levels. Furthermore, the N-terminal, but not the C-terminal Drosha can be acetylated by multiple acetyl transferases including p300, CBP and GCN5. Acetylation of Drosha competes with its ubquitination, inhibiting the degradation induced by the ubiquitin-proteasome pathway, thereby increasing Drosha protein levels. Infection of the gastric mucosa AGS cells by *H. pylori*, the gastric cancer associated carcinogen, leads to the ubiquitination and reduction of Drosha protein levels. *H. pylori* infection of AGS cells has no significant effects on Drosha mRNA levels. Our findings establish a central mechanism of protein homeostasis as playing a critical role in miRNA biogenesis.

## Introduction

MicroRNAs (miRNAs) are a class of short non-protein coding RNAs with length of ∼22 nt which impact protein expression by targeting complementary mRNA for degradation or translational inhibition. MiRNAs play important regulatory roles in myriad cellular functions such as stem cell self-renewal and cancer development [1]–[5]. The biogenesis of miRNAs is a multistep process that is initiated in the nucleus by the RNase III enzyme Drosha [6], [7]. Drosha and DiGeorge Syndrome Critical Region gene 8 (DGCR8) form a microprocessor complex to generate precursor-miRNA (pre-miRNA) by cleaving primary miRNA transcripts [8]–[11]. Pre-miRNA is subsequently transported out of the nucleus by exportin-5 in a Ran guanosine triphosphate-dependent manner for further processing into mature miRNA species by Dicer, another RNase III enzyme [12]–[15].

While a great deal of knowledge has accumulated on the roles of individual miRNA in human health and disease, little is known regarding cellular mechanisms, if any, that globally regulate miRNA synthesis. Such insight is critical given that certain disease states such as cancer appear to be associated with widespread decrease in miRNA expression [16]. Additionally, numerous disease processes appear to be associated with altered levels of proteins involved in miRNA biogenesis [17]–[20]. For example, Drosha expression is significantly decreased in 51% of ovarian cancer specimens and in specific subgroups of breast and endometrial cancers [18]–[20]. The molecular mechanisms regulating the expression levels of Drosha, however, are mostly unclear.

We report here our unanticipated finding that Drosha protein levels are increased in various cells including HEK293T, AGS and HeLa treated with deacetylase inhibitors such as trichostatin A (TSA) or nicotinamide (NIA). Using a series of Drosha expression constructs, we show that N-terminal lysines act as molecular targets that regulate Drosha protein levels by serving as substrates for the opposing forces of ubiquitination and acetylation. Multiple acetyl transferases are involved and acetylation appears to preserve Drosha protein levels by preventing ubiquitination.

## Materials and Methods

### Cell Culture and Transfection

AGS cells (ATCC) were cultured in Ham’s F-12 medium (ATCC) plus 10% fetal bovine serum (FBS) (Invitrogen). HEK293T and Huh-7 cells (ATCC) were cultured in Dulbecco’s Modified Eagle’s medium (Invitrogen) with 10% FBS and 2 mM L-glutamine (Lonza). HeLa cells (ATCC) were grown in Eagle’s Minimum Essential Medium (Lonza) supplemented with 10% FBS, 2 mM L-glutamine and non-essential amino acids (Lonza). MCF-7 cells (ATCC) were grown in Eagle's MEM, supplemented with 10% FBS, non essential amino acids (0.1 mM), 10 ug/mL Insulin (Cell Applications) and 1 mM sodium pyruvate (Mediatech). Cells were trypsinized with typsin-EDTA (Mediatech) and reseeded in culture plates 1 day before transfection. HEK293T transfection was performed with Lipofectamine (Invitrogen) when cell confluency was ∼60%. AGS cells were transfected with GenJet Plus DNA Transfection Reagent (SignaGen Laboratories) when cell confluency was ∼70%. GFP-Drosha construct has been described previously [21] and was used as a backbone for all truncated and mutated constructs. All mutants were constructed by means of site-directed mutagenesis with *pfu* DNA polymerase (Stratagene) as previously described [21], [22].

### Primary Antibodies and Agarose Beads

Drosha (D28B1) rabbit mAb (anti-Drosha), Dicer rabbit Ab, aceylated-lysine mouse mAb (ace-K) and lysine 48 linkage specific polyubiquitin antibody (K48 linked polyubiquitin) were purchased from Cell Signaling Technology; GAPDH (0411) mouse monoclonal antibody, GAPDH (FL-335) rabbit polyclonal antibody, c-Myc (9E10) mouse monoclonal antibody, c-Myc (A-14) rabbit polyclonal antibody, GFP (FL) rabbit polyclonal antibody, GFP (B-2) mouse monoclonal antibody, Exportin-5 (A-11) mouse monoclonal antibody, β-actin (R-22) rabbit polyclonal antibody, FNDC3B rabbit polyclonal antibody and protein G plus-agarose beads were purchased from Santa Cruz Biotechnology; DGCR8 rabbit polyclonal antibody was purchased from Proteintech Group.

### Western Blotting

Cell lysates were separated by 10% SDS–PAGE electrophoresis and electroblotted to nitrocellulose membrane (Bio-Rad). Blotted membranes were probed with their respective primary antibodies, rotating at 4°C overnight. Membranes were washed three times in TBST buffer and probed with secondary antibody (Alexa Fluor 680 goat anti-rabbit IgG or IRDye800-conjugated Affinity Purified Anti-Mouse IgG, respectively) at room temperature for 1 h. Membranes were then washed three times in TBST buffer and direct infrared fluorescence detection was performed with a Licor Odyssey® Infrared Imaging System [22].

### *In vitro* Acetylation Assay

HEK293T cells transfected with GFP-Drosha1–390 or GFP-Drosha391-1374. Forty-eight hours post-transfection, cell lysates were prepared with IP Lysis Buffer (Pierce). GFP antibody conjugated sepharose beads (Abcam) were used to immunoprecipitate GFP-Drosha fusion proteins. After 3 cycles of centrifugation and washing, immunoprecipitates were co-incubated with p300 (Millipore) and acetyl CoA (Sigma) at 37°C for I hr. Twenty microliters of reaction product was used for western blot.

### LC-MS/MS Mass Spectrometry Analysis

GFP-Drosha was cotransfected with CBP into HEK293T cells and incubated for 48 h. Six hours before harvesting the cells, 1 µM trichostatin A (TSA) (Sigma) was added to cell culture. Immunoprecipitated GFP-Drosha protein was resolved by 10% SDS-PAGE and visualized using Coomassie blue stain. Bands of the above protein were excised and subjected to mass spectrometry analysis as described previously [22].

### Luciferase Assays

We used the psi-CHECK2 system (Promega) to create sensor assays for quantifying mature miRNA-143 function by placing the anti-sense sequence of miRNA-143 in the 3′UTR of the gene encoding Renilla luciferase. In the presence of mature miRNA-143, the luciferase activity of Renilla decreases through the classical RNAi pathway [21]. HEK293T cells were transfected with the miR-143 psiCHECK2. Twenty-four hours post/Acetylation of Drosha Blocks/tgciq-transfection, the cells were treated with or without 2 µM Trichostatin A (TSA) for 6 hr. Firefly and Renilla luciferase activities were quantified using the Dual-Luciferase Reporter Assay System (Promega) and Renilla luciferase activity was normalized to firefly luciferase activity. For each experiment, a control employing an empty vector was used and corrected luciferase values were averaged, arbitrarily set to a value of ‘1’ and served as a reference for comparison of fold-differences in experimental values.

### Semiquantitative RT- PCR

AGS cells were treated with *H. pylori* at a 100∶1 ratio *(bacterium: cell)* for 0,1,3,6,12 and 24 h respectively. Total mRNA was extracted with TRIZOL reagent (Invitrogen) and 1 µg of total mRNA was used for cDNA synthesis using MMLV reverse transcriptase (New England Biolabs) as described in the manufacturer’s manual. 1.5 µl of cDNA was used for PCR reaction to check Drosha gene expression (Drosha Primer S: TCCAGTCATGCCGCAG; Drosha Primer A: GTGCCTGTGGTCATCATA). To measure the mRNA levels of transfected GFP-Drosha wt and mutants, total RNA was extracted from HEK293T cells transfected with respective plasmid. 1 µl of cDNA was used for PCR reaction to check mRNA levels of Drosha wt or mutants (GFP Primer S: GCAAGGGCGAGGAGC; GFP Primer A: GGTGCCTGTGGTCATCATAG). GAPDH was used as control for normalization. All PCR reactions were run for 28 cycles in triplicate and products were visualized on 1% agarose gel. As for measuring miR-143 levels in HEK293T cells treated with vehicle control or TSA overnight, TaqMan real time RT-PCR microRNA detection kits (Applied Biosystems) that include RT primers and TaqMan probes were used to quantify the levels of mature miR-143 and 18S RNA was used for normalization. All PCR reactions were run in triplicate.

### Statistical Analysis

Quantitative data were analyzed by unpaired Student’s *t* test.

## Results

### Acetylation Stabilizes Drosha Protein

**Figure 1:**
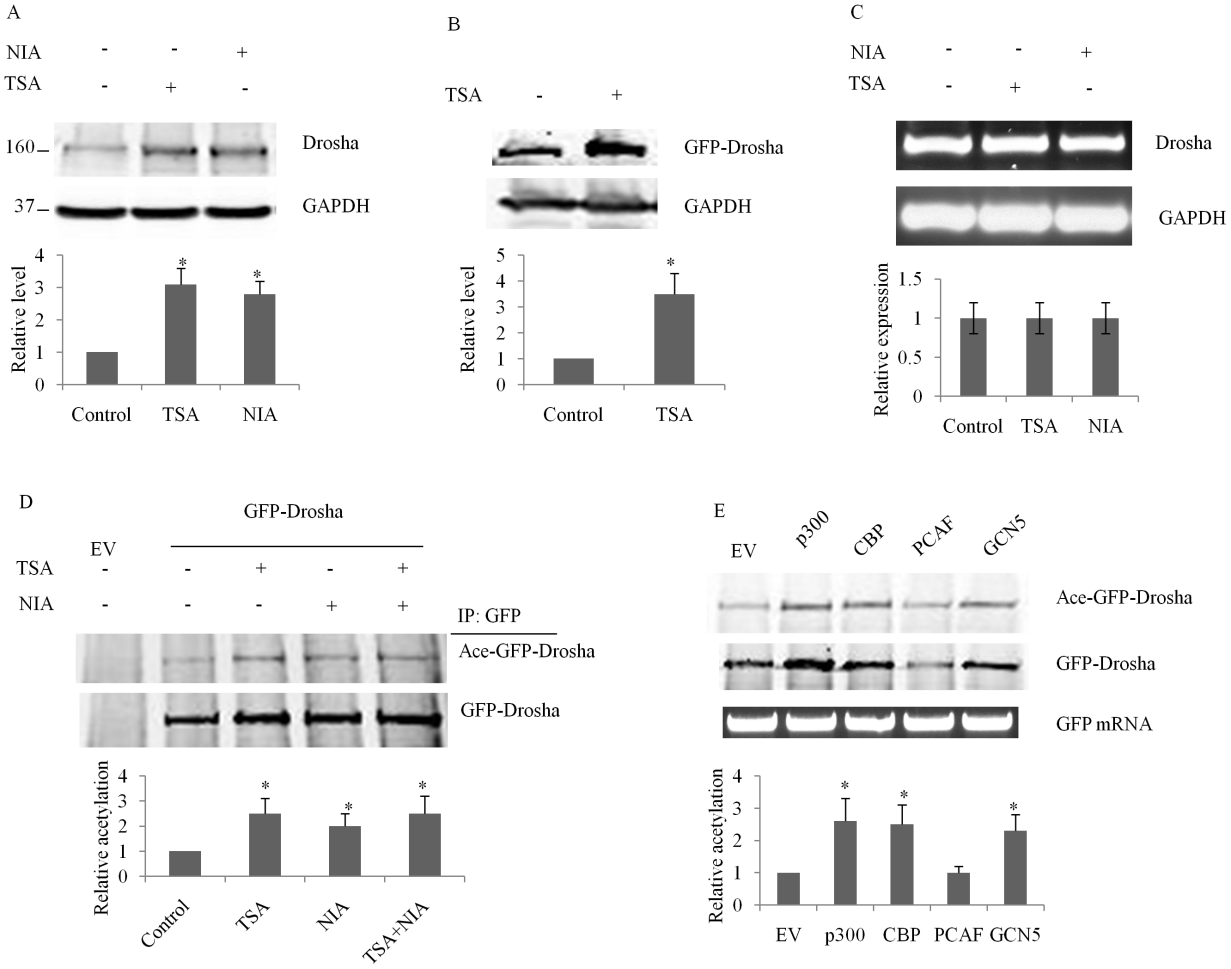
Acetylation of Drosha increases its protein level. All experiments were repeated three times with similar results (p<0.05 by Student’s t-test). (**A**), Inhibition of deacetylases increases Drosha protein level measured by western blot. HEK293T cells were treated with vehicle, trichostatin A (TSA, 2 µM), or nicotinamide (NIA, 1 mM) overnight prior to harvest. (**B**) TSA, an HDAC inhibitor, increases ectopically expressed Drosha levels in HEK293T upon transfection with a GFP-Drosha construct. (**C**) Inhibition of deacetylases have no effects on Drosha mRNA level measured by RT-PCR. (**D**)Inhibition of deacetylases increases Drosha acetylation. HEK293T cells were transfected with empty vector (EV) or GFP-Drosha. Twenty-four hours post-transfection, the cells were treated with TSA (2 µM) or NIA (1 mM) overnight. Whole cell lysates were prepared and immunoprecipitated with GFP antibody conjugated sepharose beads. The immunoprecipitates were resolved and blotted with mouse monoclonal acetylated lysine antibody to detect Drosha acetylation. The same membrane was then reblotted to check Drosha protein level. (**E**) Multiple acetyl transferases acetylate Drosha. A GFP-Drosha construct was cotransfected with an empty vector, p300, CBP, PCAF or GCN5 construct respectively into HEK293T cells. Forty-eight hours post-transfection, half of the cells was used to extract total RNA for checking the mRNA levels of GFP-Drosha. Another half of the cells was used to prepare whole cell lysates for detecting Drosha acetylation as in Figure1C.

Lysine residues serve as substrates for acetylation leading to overall stabilization of proteins [22]–[25]. To determine whether a similar dynamic existed for Drosha, we quantified protein levels in HEK293T cells after inhibition of deacetylases with trichostatin A (TSA) or nicotinamide (NIA). As seen in Figure 1A, inhibition of deacetylation lead to 3-fold increased endogenous Drosha protein levels indicating that acetylation was indeed involved in the regulation of Drosha protein stability. TSA had similar effect on exogenously expressed GFP-Drosha protein level (Figure 1B). Deacetylase inhibitors had no effects on Drosha mRNA levels (Figure 1C). We then examined the acetylation status of Drosha by western blotting and found that Drosha was constitutively acetylated and treatment with TSA or NIA further enhanced its acetylation (Figure 1D). To determine which acetyl transferase(s) were involved, we cotransfected GFP-Drosha with empty vector (EV), p300, CBP, PCAF or GCN5, respectively. The acetylation status and protein level of Drosha were determined by Western blot 48 hr posttransfection. In the meantime, GFP-Drosha mRNA levels were also measured by RT-PCR to rule out the effects of transfection efficiency on protein expression. We found that Drosha could be acetylated by multiple acetyl transferases including p300, CBP and GCN5 (Figure 1E).

### N-terminus of Drosha is the Main Region for Acetylation

**Figure 2:**
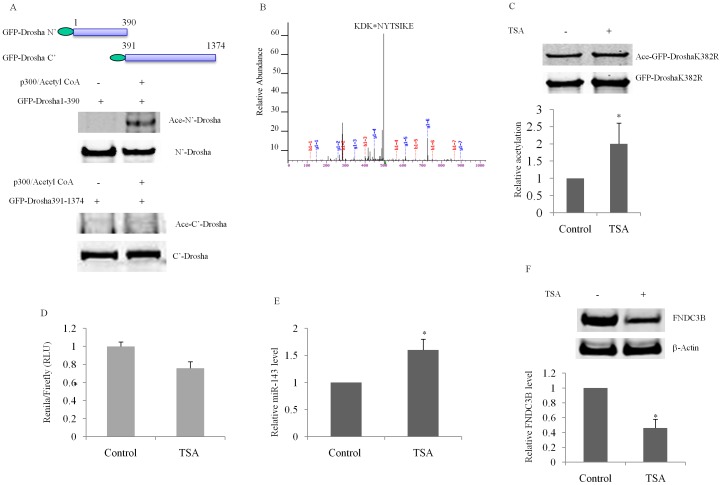
N-terminus of Drosha is the main part for acetylation. (**A**) *In vitro* acetylation assays revealed that N’-terminal but not C’-terminal Drosha is the major acetylation domain. Immunoprecipitated N-terminal Drosha (GFP-Drosha1-390 ) or C-terminal Drosha (GFP-Drosha391-1374) was co-incubated with 10 U of p300 HAT Domain and 2 µM Acetyl CoA at 37°C for I hr. (**B**) Identification of an acetylated lysine site by mass spectrometry. (C) TSA treatment increases the acetylation and expression levels of GFP-DroshaK382R (D) miRNA sensor assays revealed that compared to vehicle control, treatment of cells with TSA increased miRNA function. (E) TSA treatment increases miR-143 level in HEK293T cells. (F) TSA treatment decreases expression of fibronectin type III domain containing 3B (FNDC3B), a target of miR-143. All experiments were performed in triplicate (p<0.05 by Student’s t-test).

To identify which part of Drosha is the target for acetylation, we created both a Drosha N-terminal construct (GFP-Drosha1-390) and a Drosha C-terminal construct (GFP-Drosha 391-1374). We transfected these two constructs into HEK293T cells separately and immunoprecipitated the N-terminal and C-terminal Drosha proteins. Using these immunoprecipitated Drosha proteins as substrates to incubate with purified p300 and acetyl CoA, we found that the N-terminal Drosha1-390, but not the C-terminal Drosha 391-1374 construct, harbored the likely lysine substrates for acetylation (Figure 2A). To identify which lysines were acetylated, we immunoprecipitated Drosha protein and analyzed protein modifications by mass spectrometry. We identified lysine 382 (K382) was acetylated (Figure 2B). However, when K382 was mutated, GFP-DroshaK382K could still be acetylated (Figure 2C), indicating that multiple lysines are likely to be involved in acetylation since there are 13 lysines on the N-terminal Drosha. We note that our ability to identify one acetylation site among the multiple lysines may be due to the possibility of ion suppression of the necessary fragmented ion during targeted MS/MS analysis. To determine if acetylation of Drosha affects microRNA functions, we used a miRNA-143 psiCHECK2 construct as described previously(21). We detected increased mature miRNA-143 as shown in the form of decreased Renila luciferase activity in HEK293T cells that were transfected with psiCHECK2 and then treated with 2 µM TSA for 6 hr (Figure 2D). To further examine the effects of Drosha acetylation on microRNA processing, miR-143 level was measured by real time PCR using a miR detection kit. Compared with vehicle control, TSA treatment increased miR-143 level significantly (Figure 2E). Previous study showed that miR-143 targets fibronectin to enhance hepatocarcinoma metastasis [26]. Our result showed that TSA decreased fibronectin type III domain containing 3B (FNDC3B) evidently (Figure 2F), suggesting that acetylation of Drosha is functional in microRNA biogenesis.

### Ubiquitin-proteasome Pathway is Involved in Drosha Degradation

**Figure 3:**
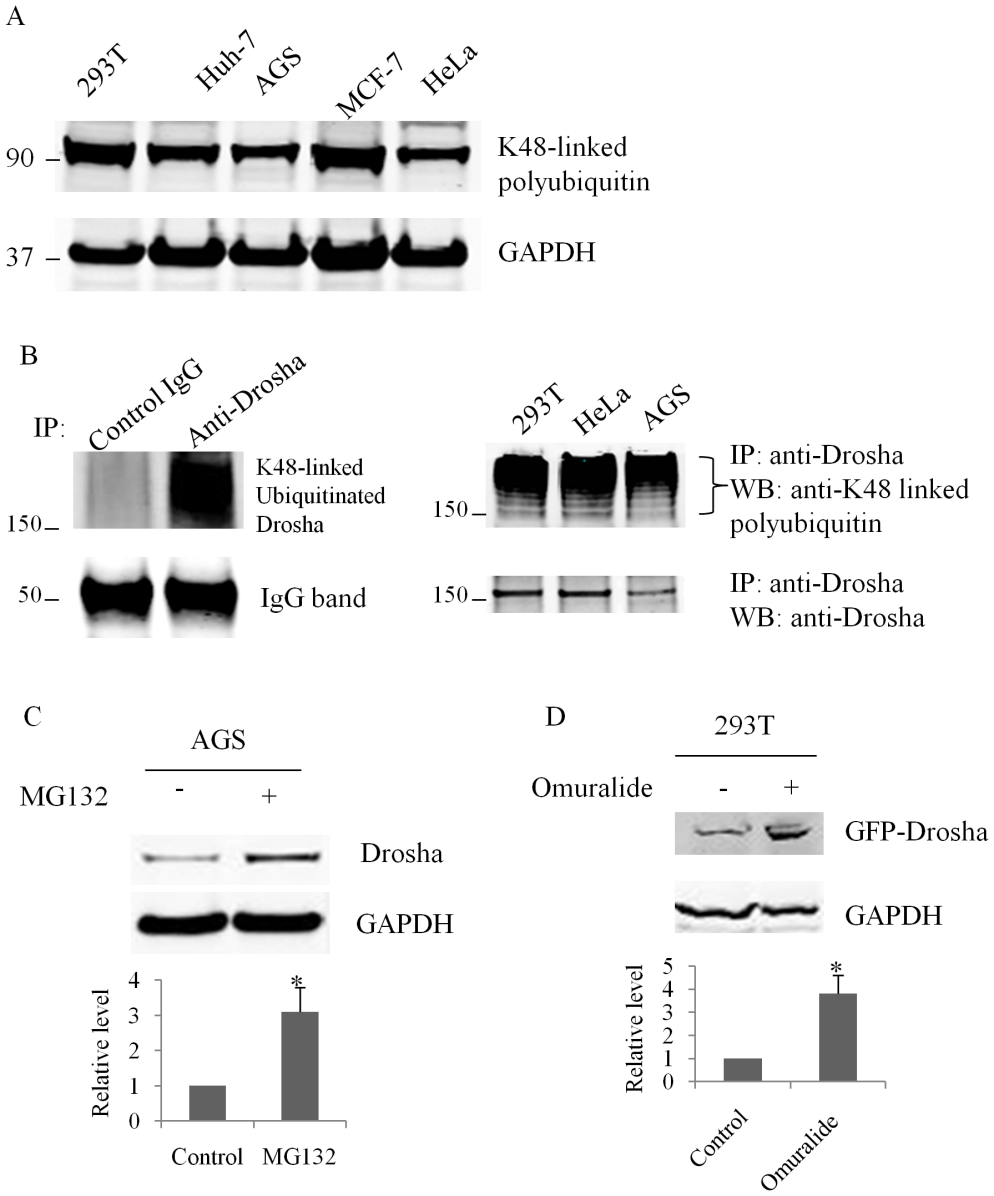
A constitutively active ubiquitin-proteasome pathway degrades Drosha. Whole cell lysates of the indicated cell types were prepared with IP Lysis Buffer (Pierce); 20 µg protein aliquots were used for western blots. All experiments were repeated three times with similar results (p<0.05 by Student’s t-test). (**A**) Abundant polyubiquitination was observed in multiple cell types using lysine 48-linkage specific polyubiquitin antibody. (**B**) Drosha is ubiquitinated in various cell types including HEK293T, HeLa and AGS. Left panel: HEK293T lysates were immunoprecipitated with control IgG or Rabbit Polyclonal Antibody to Drosha respectively. Right panel: Cell lysates were immunoprecipitated respectively with Rabbit Polyclonal Antibody to Drosha. The immunoprecipitates were resolved by SDS-PAGE and blotted with lysine 48-linkage specific polyubiquitin antibody. The same membrane was reblotted with Drosha Rabbit mAb. (**C**) Inhibition of the ubiquitin-proteasome pathway with MG132 (10 µM) increases endogenous Drosha protein level in AGS cells. GAPDH was used as a loading control. (**D**) Proteasomal inhibition increases exogenous GFP-Drosha expression level. HEK293T cells were transfected with GFP-Drosha. Twenty-four hours post-transfection, the cells were treated with 1 µM Clasto-Lactacystin-β-lactone (Omuralide) overnight. GFP-Drosha protein level was increased 3-fold following treatment.

Previous studies have shown that acetylation inhibits ubiquitination of some proteins by competing for the same lysines [24], [25]. The linking of lysine 48 (K48) of ubiquitin to substrate proteins initiates proteasomal degradation and K48-linked polyubiquitin represents the active state of the ubiquitin-proteasome pathway [27], [28]. To determine the activity of ubiquitin-pathway, we first examined K48 linked polyubiquitin levels in a wide range of cells including HEK293T, Huh-7, AGS, MCF-7 and HeLa cells. We found that this pathway was constitutively active in all the cell lines tested (Figure 3A). We also found that Drosha was heavily ubiquitinated via K48-linked polyubiquitin (Figure 3B). Treatment of AGS cells with a specific proteasome inhibitor MG132 increased Drosha protein levels 3-fold (Figure 3C). Similar increases in Drosha protein were observed upon treatment of GFP-Drosha transfected HEK293T cells with the proteasome inhibitor Clasto-Lactacystin-β-lactone (Omuralide) (Figure 3D). All of these data indicate that ubiquitin-proteasome pathway is involved in Drosha degradation.

### Multiple Lysine Residues on N-terminal Drosha are the Targets of the Ubiquitin-proteasome Pathway

**Figure 4:**
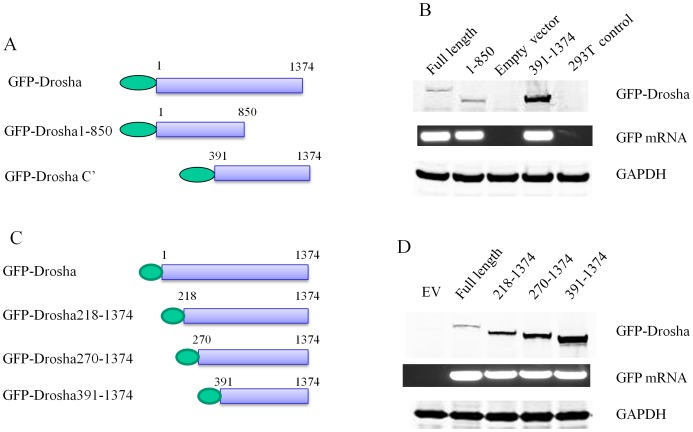
The N-terminus of Drosha is responsible for regulating its degradation. HEK293T cells were transfected with an empty vector, GFP-Drosha wild type or the various mutants as indicated. Forty-eight hours post-transfection, whole cell lysates were prepared for western blot. GAPDH was used as a loading control. All experiments were repeated three times with similar results. (**A&C**) Schematic illustration of domain deletion constructs of Drosha-GFP at the N-terminus. (**B**) GFP-Drosha 391-1374 expressed more protein than GFP-Drosha or GFP-Drosha1-850 indicating N-terminal Drosha harbors a domain regulating expression levels. The mRNA levels of GFP Drosha wt and mutants were measured to monitor the transfection efficiency of various constructs. (**D**) Deletion of additional lysines increased Drosha protein expression, indicating that multiple lysines on the N- terminus regulate Drosha degradation. The mRNA levels of GFP Drosha wt and mutants were measured to monitor the transfection efficiency of various constructs.

As mentioned, the basis of proteasomal degradation is the linkage of ubiquitin moieties to target lysine. To better define the molecular anatomy of Drosha and identify lysine motifs responsible for its stability, we created a series of GFP-tagged truncated expression constructs in which we deleted either the N-terminal 390 amino acids or C-terminal 524 amino acids (Figure 4A). We found that the N-terminal truncated mutant expressed at higher levels (∼6-fold) than the C-terminal truncated mutant (Figure 4B), suggesting that the N-terminus with its 13 lysines harbored a major regulatory domain (57 lysines in C-terminus). We next made a series of expression constructs by progressively shortening the length of N-terminal Drosha (Figure 4C). Our results revealed that the shortest construct (984aa) with the fewest lysines (n = 57) expressed at ∼6-fold higher levels compared to the full length parental construct (Figure 4D), indicating that the N-terminus of Drosha is the main part for ubiquitination.

### Acetylation and Ubiquitination Compete for Lysines on Drosha

**Figure 5:**
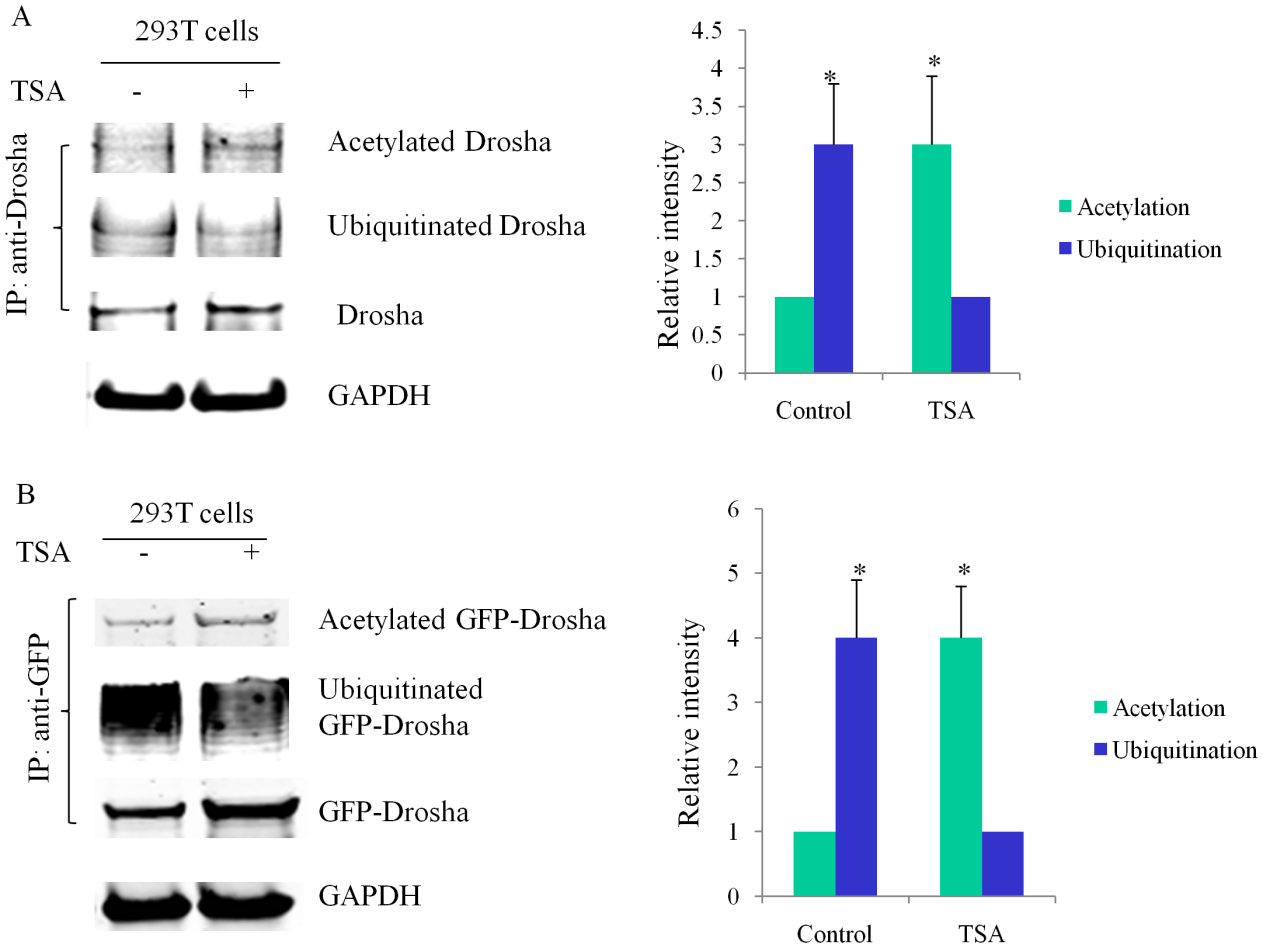
Acetylation and Ubiquitination regulate Drosha protein level. All experiments were repeated three times with similar results (p<0.05 by Student’s t-test). (**A**) Acetylation of endogenous Drosha decreases its ubiquitination. HEK293T cells were treated with or without 2 µM TSA overnight. Whole cell lysates were prepared with IP lysis buffer and 300 µg protein was immunoprecipitated with rabbit polyclonal antibody to Drosha. The immunoprecipitates were resolved and levels of acetylated Drosha were measured. The same membrane was reblotted with lysine 48-linkage specific polyubiquitin antibody to check the ubiquitination status of Drosha. A parallel IP experiment was performed to check Drosha pull down. GAPDH was used as a loading control in another parallel western blot. (**B**) TSA blocks the ubiquitination of exogenously expressed GFP-Drosha. HEK293T cells were transfected with a GFP-Drosha construct. Twenty-four hours post-transfection, the cells were treated with or without 2 µM TSA overnight. GFP antibody conjugated sepharose beads were used to immunoprecipitate GFP fusion proteins.

Treatment of HEK293T cells with deacetylase inhibitor not only increased Drosha acetylation and protein levels, but also simultaneously decreased Drosha ubiquitination (Figure 5A,) suggesting that acetylation and ubiquitination compete for the same lysines on Drosha. We further confirmed this competing effect with over-expressed GFP-Drosha (Figure 5B). We repeated the experiments in various cell lines including Huh-7, AGS and HeLa cells with similar results (data not shown).

### *H. pylori* Infection Facilitates Drosha Degradation through Enhancing Ubiquitin-proteasome Pathway

**Figure 6:**
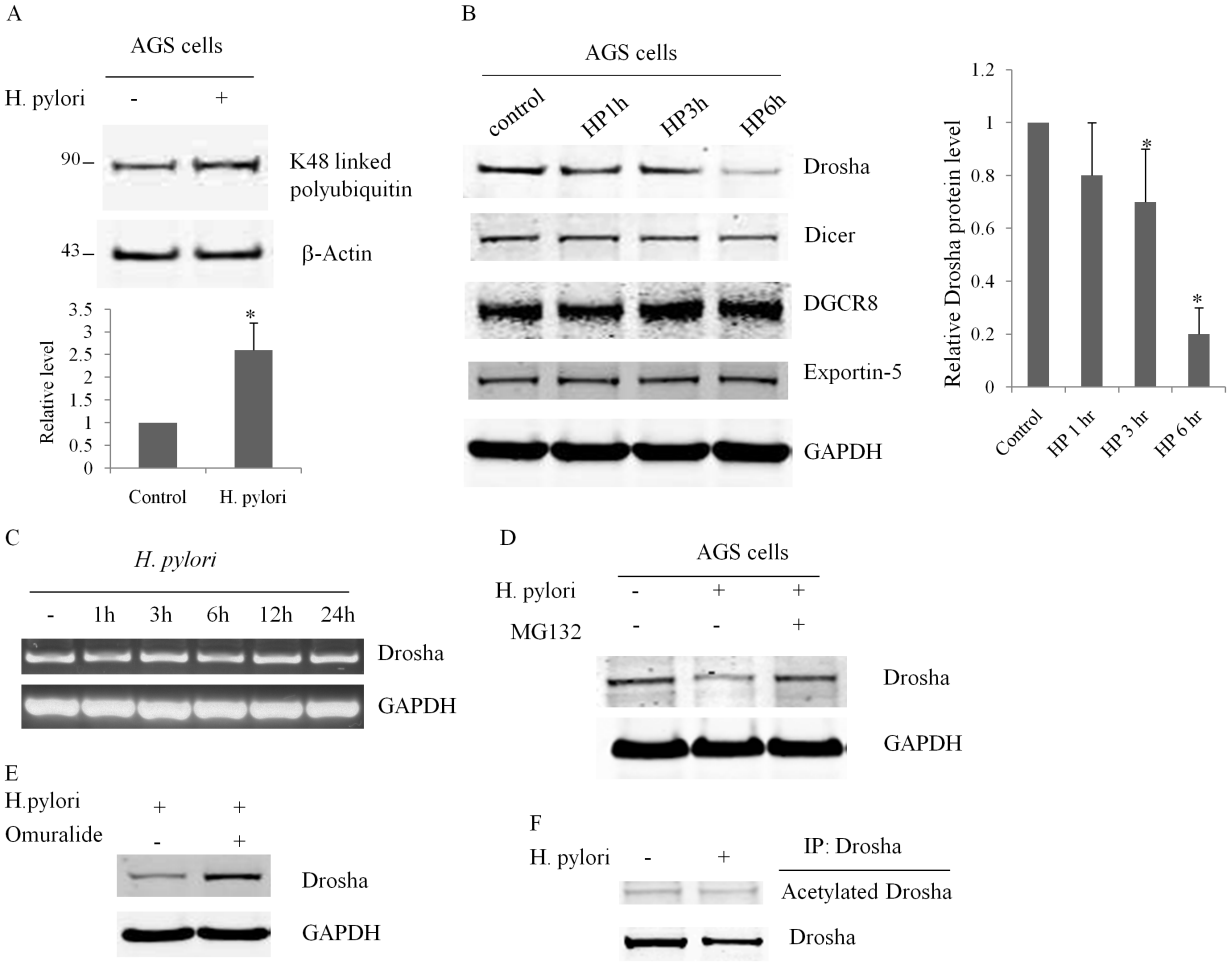
*H. pylori* infection degrades Drosha through the ubiquitin-proteasome pathway. AGS gastric epithelial cells were infected with *H. pylori* at a ratio of 100∶1 (bacterium:cell) for different time periods. All experiments were repeated three times with similar results (p<0.05 by Student’s t-test). (**A**) *H. pylori* increased levels of K48-linked polyubiquitin. AGS cells were infected with *H. pylori* for 6 h. Beta-Actin was used as a loading control. (**B**) Western blot revealed that Drosha protein is decreased by *H. pylori* infection. GAPDH was used as a loading control. Other miRNA related proteins were not affected. (**C**) *H. pylori* infection does not alter Drosha mRNA levels. Total mRNA from AGS cells was reverse transcribed and amplified using Drosha specific primers. Gelstar was used to stain PCR products. (**D**) MG132, a specific proteasome inhibitor, rescued Drosha from *H. pylori* induced degradation. AGS cells were infected with *H. pylori* for 6 h and GAPDH was used as a loading control. (**E**) Omuralide, another specific proteasome inhibitor, also rescued Drosha from *H. pylori* induced degradation. AGS cells were infected with *H. pylori* for 6 h and GAPDH was used as a loading control. (**F**) H. pylori infection doesn’t affect Drosha acetylation.

Our earlier work had demonstrated that *H. pylori* infection accelerated p27 degradation via a proteasome-dependent pathway [29]. Infection of human gastric epithelial AGS cells with wild-type *H. pylori* strain 60190 (ATCC49503) at a 100∶1 ratio *(bacterium: cell)* significantly increased K48 linked polyubiquitin, indicating that the ubiquitin-proteasome pathway was activated by *H. pylori* (Figure 6A). We measured expression levels of miRNA-associated proteins including Drosha, Dicer, DGCR8, Exportin-5 by western blotting. Surprisingly, Drosha protein levels were decreased by 80% after *H. pylori* infection for 6 hours (Figure 6B). Drosha mRNA levels were not affected, suggesting that *H. pylori* infection influenced Drosha expression levels post-transcriptionally (Figure 6C). *H. pylori* infection had no effect upon the other miRNA-related proteins examined (Figure 6B). To further confirm the activation of ubiquitin-proteasome pathway by *H. pylori*, we treated AGS cells with a specific proteasome inhibitor (MG132) and found that Drosha protein levels were preserved despite *H. pylori* infection and approached levels found in uninfected cells (Figure 6D). Omuralide had similar effect as MG132 (Figure 6E). *H. pylori* infection had no appreciable effect on Drosha acetylation (Figure 6F).

## Discussion

MiRNA biogenesis is a step-wise process involving multiple proteins and precedence exists for post-translational modifications impacting the expression and function of miRNA related proteins [30]. For example, HDAC1 enhances microRNA processing via deacetylation of DGCR8 [31]. Phosphorylation of TRBP at S142, S152, SS283 and S286 by the MAP kinase Erk stabilizes this protein and enhances miRNA production [32]. Ubiquitination of Ago2 by mLin41 targets Ago2 for proteasomal degradation [33]. With respect to Drosha, phosphorylation of Ser 300/302 facilitates its localization to the nucleus [21], [34]. Here, we began with our observation of an unexpected increase in Drosha protein following treatment of HEK293T cells with deacetylase inhibitors. *In silico* screening suggested that several lysines on the N-terminus of Drosha could be the targets for ubiquitination or acetylation. Using a series of truncated and mutated Drosha constructs, we confirmed that N-terminus of Drosha habors a major region containing lysines which can be ubiquitinated or acetylated. These mutually exclusive post-translational modifications have opposing effects of degradation and stabilization, respectively, and suggest that N-terminal centered lysines serve as molecular targets mediating Drosha protein expression. This regulation of protein stability is reminiscent of that observed for other proteins: acetylation of p53 inhibits its ubiquitination [24] and a similar mechanism exists for WRN protein [25].

Our previous study showed that increase of Drosha expression level dramatically facilitates miRNA-143 expression [21]. Treatment of HEK293T cells with Trichstatin A (TSA), a specific histone deacetylase (HDAC) inhibitor, increases Drosha protein level up to 3-fold (Figure 1 A & B). However, in a microRNA sensor assay, TSA treatment increased miRNA-143 function in a modest but statistically significant manner (Figure 2D). A recent report by Wada et al lends an explanation to this seeming inconsistency. Wada et al found that acetylation of DGCR8 inhibits its binding with pri-miRNA; HDAC1 enhances miRNA processing via deacetylation of DGCR8 [31]. Initiation of miRNA processing by microprocessor requires the coordination of Drosha and DGCR8. Inhibition of HDAC1 by TSA hinders DGCR8 function, offsetting a large part of facilitating Drosha function.

In some human diseases, the ubiquitin-proteasome pathway may be disrupted. For example, in colon and renal cell cancers, the ubiquitin ligases are coded by oncogenes [35]. In *H. pylori*-infected gastric epithelial cells, the proteasome-mediated degradation of the cyclin-dependent kinase inhibitor p27^kip1^ is enhanced [29]. Accumulating evidence shows that overall miRNA levels in gastric epithelial cells are decreased in *H. pylori* infected patients [36]. Our work suggests that *H. pylori* infection enhances the ubiquitin-proteasome pathway which is constitutively active in gastric epithelial cells. Of the miRNA regulatory proteins, Drosha appears far more sensitive to ubiquitin-proteasome pathway induced degradation than Dicer, DGCR8 or exportin-5. Activated ubiquitin molecules are ligated to the N-terminal lysines of Drosha leading to its degradation. Those lysine residues are also the acetylation sites of multiple acetyl transferases such as p300, CBP. Acetylation of Drosha on those N-terminal lysine sites prevents Drosha ubiquitination, thereby preventing its degradation. In conclusion, our findings clarify cellular mechanisms impacting Drosha protein levels and provide a mechanism by which *H. pylori* alters miRNA expression and downstream global gene regulation. Our discoveries also indicate a mechanistic link between chronic *H. pylori* infection and malignant transformation of gastric cells. These findings implicate a potential therapeutic insight for mitigating the influence of long-term *H. pylori* infection on its adverse clinical sequelae, including gastric malignancies [37].
